# Treatment intervals with first-generation anti-vascular endothelial growth factor drugs: evaluating the unmet need in a real-world neovascular age-related macular degeneration national database

**DOI:** 10.1038/s41433-025-03996-8

**Published:** 2025-10-16

**Authors:** Javier Zarranz-Ventura, Gonzaga Garay-Aramburu, Pilar Calvo, Miguel Angel Zapata, Carolina Arruabarrena, Pablo Arnáiz, Paula García-Lunar, Laura Sararols-Ramsay

**Affiliations:** 1 Hospital Clínic de Barcelona, Universitat de Barcelona, Barcelona, Spain; 2 Fundació Clínic per a la Recerca Biomedica – Institut de Investigacions Biomediques August Pi i Sunyer (FCRB-IDIBAPS), Barcelona, Spain; 3 Begiker-Ophthalmology Research Group, Department of Ophthalmology, Biocruces Bizkaia Health Research Institute, OSI Bilbao Basurto, Facultad de Medicina, Campus de Basurto, University of the Basque Country, UPV/EHU, Bilbao, Spain Spain; 4 Miguel Servet Ophthalmology Research Group (GIMSO), Miguel Servet University Hospital, Aragón Institute for Health Research (IIS-Aragón), Zaragoza, Spain; 5 Hospital Universitari Vall de Hebrón, Barcelona, Spain; 6 Hospital Universitario Príncipe de Asturias, Madrid, Spain; 7 Roche Farma, Madrid, Spain; 8 Fundació Privada Hospital Asil Granollers, Granollers, Spain; 9 Hospital Clínic de Barcelona, Barcelona, Spain; 10 Hospital Universitario Vall de Hebrón, Barcelona, Spain; 11 Hospital Universitario del Henares, Madrid, Spain; 12 Hospital Universitario Miguel Servet, Zaragoza, Spain; 13 OSI Basurto, Bilbao, Spain; 14 OSI Araba, Vitoria, Spain; 15 Hospital de Conxo, Santiago de Compostela, Spain; 16 Hospital Universitario de La Princesa, Madrid, Spain; 17 Hospital Universitario Ramón y Cajal, Madrid, Spain; 18 Hospital Consorci Sanitari Moises Broggi, Hospitalet del Llobregat, Barcelona, Spain; 19 Hospital Universitario Puerta del Hierro, Madrid, Spain; 20 Hospital de Torrevieja, Alicante, Spain; 21 Hospital Punta de Europa, Cádiz, Spain; 22 Clínica Universidad de Navarra, Pamplona, Spain; 23 Hospital Dos de Maig, Barcelona, Spain; 24 Hospital Universitari Germans Trias i Pujol, Badalona, Spain; 25 Hospital Universitario de Bellvitge, Hospitalet del Llobregat, Llobregat, Spain; 26 Hospital Clínico Universitario Lozano Blesa, Zaragoza, Spain; 27 Complejo Hospitalario Universitario de Ferrol, Ferrol, Spain; 28 Villoria Clinic, Pontevedra, Spain; 29 Hospital Universitario del Vinalopo, Alicante, Spain; 30 Clínica Oftalvist Valencia, Valencia, Spain; 31 Hospital Universitario La Paz, Madrid, Spain; 32 Hospital Universitario Fundación Jiménez Díaz, Madrid, Spain; 33 Hospital San Juan de Dios del Aljarafe, Sevilla, Spain; 34 Hospital Costa del Sol, Marbella, Spain; 35 Centro de Ojos de La Coruña, A Coruña, Spain; 36 Hospital Universitario Virgen de las Nieves, Granada, Spain; 37 Clínica Oftalvist Madrid, Madrid, Spain; 38 Hospital Universitario de Cruces, Bilbao, Spain; 39 Hospital del Mar, Barcelona, Spain; 40 Hospital de la Ribera de Alzira, Valencia, Spain; 41 Clínica Oftalvist Sevilla, Sevilla, Spain; 42 Clínica Rementería, Madrid, Spain; 43 Hospital Universitario Virgen del Rocio, Sevilla, Spain; 44 Hospital do Meixoeiro, Vigo, Spain

**Keywords:** Medical research, Outcomes research

## Abstract

**Background:**

To evaluate treatment intervals at 12/24 months following initiation of anti-VEGF therapy and to characterise the clinical profile of neovascular AMD (nAMD) patients achieving extended treatment intervals (≥12 and ≥16 weeks).

**Methods:**

National, retrospective, real-world study using data from the validated web-based Fight Retinal Blindness (FRB!) registry. Treatment-naive nAMD eyes managed with approved first-generation intravitreal VEGF inhibitors (ranibizumab, aflibercept 2 mg) and followed for at least 12 months were included. A subanalysis was conducted on eyes receiving a number of injections within range of a treat and extend (TAE) regimen at 12 and 24 months.

**Results:**

A total number of 1278/557 treatment-naïve nAMD eyes within the TAE range category completed the required follow up at 12/24 months. At 12 months, 39.3% of eyes remained on ≤Q8W, 22.5% >Q8W- < Q12W, 29.1% ≥Q12W- < Q16 and 9.1% ≥Q16W. At 24 months, the distribution was 35.4%, 17.6%, 28.3% and 18.7%, respectively. Mean VA change was not significantly different between groups at both 12 months (≤ Q8W: +4.7, Q8-Q12: +3.5, Q12-Q16: +6.1, ≥Q16W: +4.8 letters) and 24 months (≤Q8W: +5.8, Q8-Q12: +3.7, Q12-Q16: +4.1, ≥Q16W: +3 letters). The percentage of visits with active lesions was similar across groups at both time points, indicating consistent disease control.

**Conclusions:**

Despite receiving a number of injections within a TAE range, a substantial proportion of eyes failed to achieve extended treatment intervals at 12 and 24 months (61.8% and 52.9%, respectively). These results underscore the significant unmet therapeutic need in the management of nAMD with currently approved first-generation anti-VEGF agents.

## Introduction

Neovascular age-related macular degeneration (nAMD) is the leading cause of legal blindness in older adults in most developed countries [1]. Anti-vascular endothelial growth factor (anti-VEGF) drugs are the gold-standard treatment for nAMD [2] and have significantly contributed to reducing the prevalence of visual impairment at a population level [3, 4]. While randomised clinical trials (RCT) have demonstrated substantial improvements in visual outcomes with anti-VEGF therapies [5], these results have not been consistently replicated in real-world clinical practice [6]. This discrepancy may be attributed to several factors, including the inclusion of broader, unselected patient populations in observational studies, suboptimal treatment adherence due to the high frequency of injections required for disease control, and the limited durability of first-generation anti-VEGF agents [7–9]. These challenges contribute to a considerable treatment burden for both patients and healthcare providers, potentially compromising long-term visual outcomes.

The collection of real-world data on AMD management has been facilitated by the widespread adoption of electronic medical records systems. The Fight Retinal Blindness (FRB!) registry [10] is an international consortium health outcomes measurement (ICHOM)-compliant web-based platform that enables the systematic and rapid capture of clinically relevant data fields, thereby supporting the investigation of key clinical questions related to the treatment of nAMD [11–13]. Despite these advances, certain aspects remain relatively underexplored, particularly the durability of anti-VEGF therapies and treatment responses under extended dosing intervals in routine clinical settings. This represents a critical gap, as raw observed treatment intervals may reflect undertreatment or poor adherence rather than a favourable therapeutic response [14]. Consequently, additional inclusion criteria are required in the analysis plan to ensure that the eyes under evaluation have received appropriate disease management in routine clinical care. While this approach may reduce the external validity of the findings relative to a broader cohort, it enhances the interpretability and clinical relevance of the results.

Recent advances in anti-VEGF therapy have introduced novel second-generation agents designed to reduce injection frequency and extend treatment intervals by targeting additional molecular pathways involved in vascular stabilisation (i.e. faricimab, blocking VEGF and also angiopoietin-2) [15] or elevating the concentration of pre-existing drugs (i.e. aflibercept 8 mg, compared to the classic 2 mg) [16]. RCTs have demonstrated that both approaches effectively extend treatment intervals while maintaining visual and anatomical outcomes over 24 months [15, 16]. Moreover, dual-pathway inhibition with faricimab has shown potential for enhanced disease control, as evidenced by greater reductions in macular leakage area and retinal hyperreflective foci volume in both RCTs and real-world settings [17, 18] leading to increased durability and improved clinic capacity [19].

To assess the potential clinical benefit of these longer-acting therapies, it is essential to first characterise current treatment patterns with first-generation anti-VEGF agents in routine clinical care. Quantifying the proportion of eyes that fail to achieve extended treatment intervals provides a measure of the unmet therapeutic need and identifies candidates who may benefit from newer agents. This information is critical for informing policy decisions and guiding the integration of second-generation therapies into clinical pathways, which are influenced by local healthcare infrastructure, reimbursement models, and regulatory frameworks. These considerations are particularly relevant in the context of the emerging availability of biosimilar formulations of first-generation anti-VEGF agents.

This study aims to quantify the extent of the unmet need in achieving extended treatment intervals with approved first-generation anti-VEGF agents (ranibizumab, aflibercept 2 mg) in the management of nAMD. Specifically, it evaluates the proportion of eyes that fail to reach 12- or 16-week injection intervals at 12 and 24 months, based on data collected in the national nAMD registry.

## Methods

### Study design, setting and ethics approval

Retrospective, observational analysis (SL44438) of nAMD-treated eyes included in the FRB! registry nationwide (FRB Spain project) in routine clinical care [10, 13, 20–22]. Ethics approval was obtained from the coordinating centre Institutional Review Board (IRB) (Hospital Clinic Barcelona) and all local authorities. The study adhered to the tenets of the Declaration of Helsinki. All patients in ongoing treatment provided their written informed consent to be included in the registry.

### Data sources

Data collection was completed using the nAMD module of the FRB! registry, a validated ICHOM-compliant online web-based tool [10, 23]. Data collected included visual acuity (VA), treatment given, and ocular adverse events at baseline and at each subsequent visit. Demographic characteristics (age and sex), prior treatments (including cataract surgery and vitrectomy) or comorbidities were recorded at the baseline visit. Treatment decisions, including the choice of VEGF inhibitor and visit schedule, were driven locally at the physician’s discretion in each centre, thereby reflecting daily clinical practice. Data extraction was performed in May 2023.

### Outcomes

The primary objective of this analysis was to describe the treatment and disease burden after anti-VEGF treatment initiation by analysing the treatment intervals and the number of visits/injections at 12 months. Secondary objectives included the 24 months outcomes, the baseline clinical characteristics of treated eyes and the clinical outcomes of eyes achieving and not achieving extended intervals at both timepoints. The visit of the first anti-VEGF injection was considered the baseline visit. Months 12 and 24 visits were the visits that occurred 12 and 24 months (±1.5 months) after the first anti-VEGF injection.

### Study cohorts

Two study cohorts were evaluated, the overall cohort and the Treat and extend (TAE) cohort. Inclusion criteria for the overall cohort were nAMD adult (≥18 years) patients treated with an approved anti-VEGF (aflibercept, ranibizumab, brolucizumab) from the start of anti-VEGF treatment (considering the first anti-VEGF injection as the baseline visit, dated before 1st January 2022) and followed-up for at least 12 months (±1.5 months) after the first injection. The TAE cohort was selected as a subpopulation from the overall cohort using additional criteria, such as completion of a correct loading dose (at least 3 injections during the first 16 weeks since baseline); between 6 and 13 injections during the first year (12-months cohort), and between 8 and 24 injections during the first two years (24-months cohort); no treatment intervals greater than 20 weeks (12- and 24-month cohorts); and no gap between visits greater than 365 days during their first 24 months since baseline visit. The analysed eyes were grouped by four-week intervals according to the interval observed between the closest injection at 12 and 24 months and the previous one, in 8 weeks or less (≤ 8 weeks; ≤Q8W), more than 8 weeks but less than 12 weeks (8-12 weeks; >Q8W-<Q12W), 12 weeks or more but less than 16 weeks (≥ 12-16 weeks; ≥Q12W-<Q16W) or 16 weeks or more (≥16 weeks; ≥Q16W). These specific limits could inform suboptimal response estimations to current available drugs and highlight potential unmet needs for novel approved therapies [15, 16].

### Statistical analysis

A description of the study variables was provided. Summary statistics for categorical variables included frequency (N) and percentage (%) of each category/modality. Summary statistics for continuous variables included mean, standard deviation (SD), first quartile (Q1), median, and third quartile (Q3). For VA change, 95% confidence intervals (95% CI) are provided.

## Results

### Baseline characteristics

A total of 1950 eyes from 1629 patients were included in the overall cohort (Supplementary Table 1). From these, the TAE cohort included 1278 eyes which fulfilled the additional selection criteria and had a minimum follow up of 12 months, and 557 eyes were followed up for 24 months. This TAE cohort constituted the core of the analysis. Demographic and baseline characteristics of these patients and eyes are shown in Table 1. Briefly, mean (SD) age was 79.6 (7.6) years and 59% of patients were female. Ranibizumab was the initial anti-VEGF most frequently received (58%). Macular neovascularisation lesion type was classified in 55.8% of the study eyes (*n *= 714), and the most frequent type was type 1 (44.2%, *n *= 316) followed by type 2 (27.7%, *n* = 198), type 3 (21.0%, *n* = 150) and aneurysmatic type 1 (polypoidal choroidal vasculopathy, 5.4%, *n *= 39).

**Table 1:** Demographic and clinical characteristics of study eyes.

Variable	nAMD
Eyes (*n*)	1278
Patients (*n*)	1122
Gender, % female patients	59
Age, mean (SD)	79.6 (7.6)
Smoking status, *n* (%)	
*Active smoker*	51 (4)
*Non-smoker*	271 (21)
*Ex-smoker*	102 (8)
*Unknown*	854 (67)
Time from diagnosis (days), median (Q1, Q3)	0 (0, 6)
Baseline VA, mean (SD)	56.6 (19.5)
*≤35 letters, n (%)*	218 (17)
*≥70 letters, n (%)*	404 (32)
Lesion type (*n* = 714, 55.8%), *n* (%)	714 (100%)
*Type 1*	316 (44.2)
*Type 2*	198 (27.7)
*Type 3*	150 (21.0)
*PCV*	39 (5.4)
*Mixed*	11 (1.5)
Initial injection, *n* (%)	
*Aflibercept*	540 (42)
*Brolucizumab*	0 (0)
*Ranibizumab*	738 (58)

*nAMD* Neovascular age-related macular degeneration, *PCV* Polypoidal choroidal vasculopathy, *Q1* first quartile, *Q3* third quartile, *SD* Standard deviation, *VA* Visual acuity.

### Treatment intervals

In the overall cohort, 12 months after anti-VEGF treatment initiation 33.1% of eyes were on a ≤Q8W interval, 17.5% were on a >Q8W-<Q12W interval and 49.4% were on a ≥Q12W interval. At 24 months, 28.1% of eyes were on a ≤Q8W interval, 13.1% were on a >Q8W-<Q12W interval and 58.8% were on a ≥Q12W interval. The main results for this population are shown in Supplementary Table 2.

**Fig. 1:**
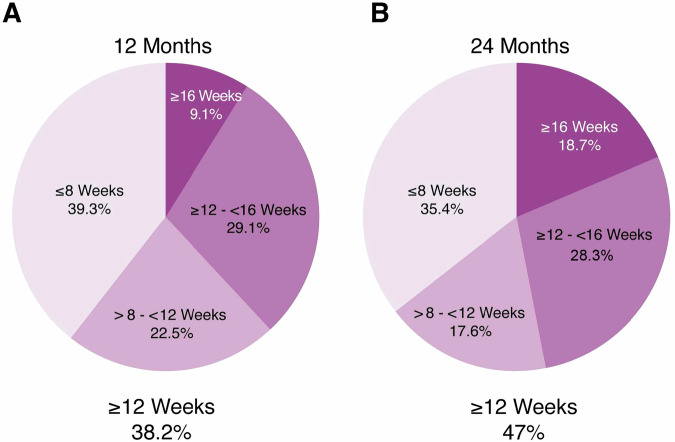
Treatment intervals at study timepoints. Percentage of eyes by last treatment interval after anti-VEGF treatment initiation at month 12 (**A**) and at month 24 (**B**). Groups included ≤Q8W, >Q8W-<Q12W, ≥Q12W-<Q16W, ≥Q16W intervals.

In the TAE cohort at 12 months of follow-up the percentage (*n*) of eyes according to their last treatment interval category was: 39.3% (*n* = 502) on a ≤Q8W interval, 22.5% (*n* = 288) on a >Q8W-<Q12W interval, 29.1% (*n* = 372) on a ≥Q12W–Q<16W interval and 9.1% (*n* = 116) ≥Q16W (≥Q12W = 38.2%) (Fig. 1A, Table 2). At 24 months of follow-up, 35.4% of study eyes (*n* = 197) were on ≤Q8W, 17.6% (*n* = 98) were on >Q8W-<Q12W, 28.3% (*n* = 158) were on ≥Q12W-<Q16W and 18.7% (*n* = 104) were on ≥Q16W (≥Q12W = 47%) (Fig. 1B, Table 2). The percentage of eyes achieving intervals ≥Q12W was 38.2% and 47% at months 12 and 24. Particularly, 9.1% and 18.7% of patients achieved intervals ≥Q16W at 12 and 24 months, respectively (Fig. 1A, B, Table 2).

**Table 2:** Summary of treatment and disease burden at 12 and 24 months after anti-VEGF treatment initiation.

Variable	12 Months	24 Months
Eyes (*n*)	1278	557
Injections, mean (SD)	8 (1.3)	13.8 (2.7)
Injections, median (Q1, Q3)	8 (7, 9)	13 (12, 15)
Visits, mean (SD)	9.2 (1.9)	15.9 (4)
Visits, median (Q1, Q3)	9 (8, 10)	15 (13, 18)
Maximum treatment interval, median (Q1, Q3)	84 (70, 98.8)	103 (85, 119)
Most frequent treatment interval category, *n* (%)		
*4 weeks*	597 (47)	152 (27)
*6 weeks*	332 (26)	138 (25)
*8 weeks*	189 (15)	129 (23)
*10 weeks*	117 (9)	73 (13)
*12 weeks*	28 (2)	32 (6)
*14 weeks*	13 (1)	23 (4)
*16 weeks*	2 (0)	8 (1)
*18+ weeks*	0 (0)	2 (0)
Last treatment interval, median (Q1, Q3)	69 (54, 84)	71 (56, 98)
Last treatment interval category, *n* (%)		
*4 weeks*	91 (7)	36 (6)
*6 weeks*	170 (13)	70 (13)
*8 weeks*	241 (19)	91 (16)
*10 weeks*	288 (23)	98 (18)
*12 weeks*	233 (18)	85 (15)
*14 weeks*	139 (11)	73 (13)
*16 weeks*	49 (4)	64 (11)
*18+ weeks*	67 (5)	40 (7)

*Q1* first quartile,* Q3* third quartile, *SD* Standard deviation, *VA* Visual acuity.

**Fig. 2:**
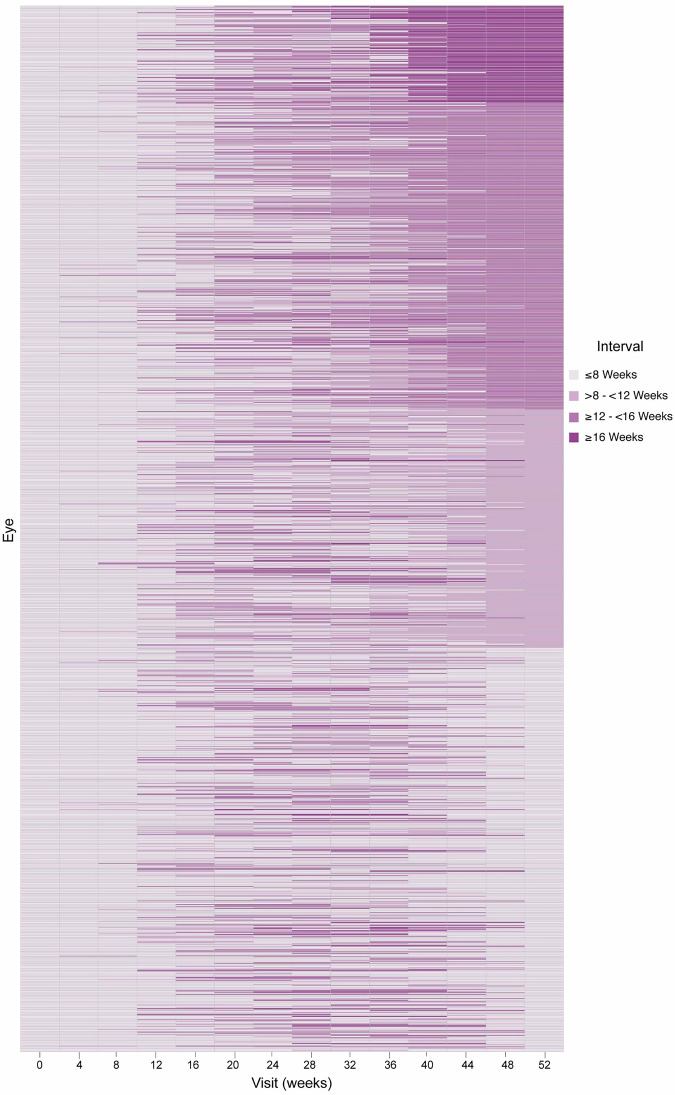
Frequency of treatment intervals during the first 12 months after anti-VEGF treatment initiation. Each column represents the treatment interval between the injection received in this visit and the previous one. The last column corresponds to the last treatment interval between injections at week 52.

**Fig. 3:**
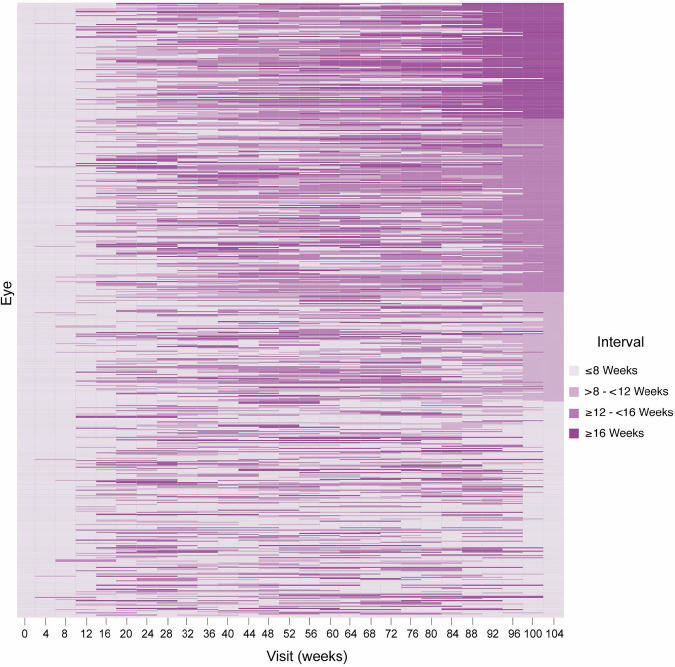
Frequency of treatment intervals during the first 24 months after anti-VEGF treatment initiation. Each column represents the treatment interval between the injection received in this visit and the previous one. The last column corresponds to the last treatment interval between injections at week 104.

The evolution of treatment intervals during the first 12 and 24 months after anti-VEGF treatment initiation in this specific population is graphically presented in Figs. 2 and 3. The most frequent treatment interval was 4 weeks both at month 12 (47%) and at month 24 (27%) followed by 6 weeks at month 12 (26%) and at month 24 (25%). Intervals shorter than Q10W (Q4W, Q6W, Q8W) were more frequent than the longer ones at both follow-up time points (Table 2). The maximum treatment interval (median, Q1-Q3) was 84 days (70–98.8) and 103 days (85–119) at 12 and 24 months, respectively.

### Number of injections and visits

In the TAE cohort, at 12 months the median (Q1, Q3) number of injections and visits was 8 (7, 9) and 9 (8, 10), and at 24 months the median number of injections and visits was 13 (12, 15) and 15 (13, 18) (Table 2). The mean (SD) number of injections/visits was 8 (1.3)/9.2 (1.9) at month 12 and 13.8 (2.7)/15.9 (4) at month 24. The number of injections and visits according to their last treatment interval category is shown in Supplementary Tables 3 and 4 (at 12 and 24 months, respectively). In the overall cohort, at 12 months the median (Q1, Q3) number of injections/visits was 7 (6, 8)/8 (7, 10) and at 24 months it was 11 (9, 14)/14 (12, 17) (Supplementary Table 2).

### Clinical outcomes according to the last treatment interval category

In the TAE cohort, the mean baseline VA (SD) was similar between groups at month 12: 57 (19), 56.8 (18.7) 56.1 (20.3) and 55.9 letters (21.3) for eyes treated ≤Q8W, >Q8W-<Q12W, ≥Q12W-<Q16W and ≥Q16W, respectively. The mean (95% CI) VA change from baseline to final evaluation was +4.7 (3.1, 6.2) for eyes treated ≤Q8W, +3.5 (1.6, 5.4) for eyes >Q8W-<Q12W, +6.1 (4.3, 7.9) for eyes on ≥Q12W-<Q16W and +4.8 (1.8, 7.8) for eyes on ≥Q16W. Similar results were observed among eyes followed for 24 months: 60.4 (17.1), 59 (17.4) 59.5 (16.4) and 59.2 (18.6) at baseline for eyes treated ≤Q8W, >Q8W-<Q12W, ≥Q12W-<16W and ≥Q16W, respectively. The mean (95% CI) VA change was +5.8 (3.5, 8.1) for eyes treated ≤Q8W, +3.7 (−0.2, 7.6) for eyes >Q8W-<Q12W, +4.1 (1.2, 7) for eyes on ≥Q12W-<Q16W and +3 (−0.3, 6.4) for eyes on ≥Q16W. Additionally, the percentage of eyes with inactive lesion activity at last visit was numerically higher than at all visits for each category at 12 months (all visits vs last visit, inactive: ≤Q8W, 19% vs 30%; >Q8W-<Q12W, 23% vs 34%; ≥Q12W-<16W, 37% vs 54%; ≥Q16W, 28% vs 43%) and at 24 months (all visits vs last visit, inactive: ≤Q8W, 18% vs 25%; >Q8W-<Q12W, 27% vs 39%; ≥Q12W-<Q16W, 35% vs 50%; ≥Q16W, 35% vs 55%) (Supplementary Table 4).

## Discussion

This study offers a robust estimation of the unmet therapeutic need associated with classic first-generation anti-VEGF drugs in a national dataset of nAMD treated eyes. By evaluating treatment intervals at 12 and 24 months, the analysis employs these intervals as surrogate indicators of the treatment burden necessary to sustain visual acuity gains under real-world clinical conditions. The findings underscore the potential value of integrating novel pharmacological agents and treatment protocols to alleviate the treatment burden in the management of nAMD at a multicentre, national level.

The results presented in this study characterise national adherence to established treatment guidelines for nAMD, as evidenced by the mean number of injections administered across different treatment interval groups. At 12 months, injections frequencies ranged from 6.8 to 8.9, and at 24 months, from 11.8 to 15.7, aligning with real-world evidence (RWE) from European studies reporting mean injection counts between 4 and 9 range at month 12 [7, 9, 13, 24, 25]. Notably, the 24-month injection rates observed in our cohort slightly exceeded the European range of 6.1 to 11, and were more consistent with those reported in Canada (12.1), Australia (13.0), or the USA (14.3) [13]. These data underscore the persistent treatment burden associated with nAMD management over time, as corroborated by extension studies [25, 26]. Furthermore, the discrepancy between visual outcomes achieved in clinical trials and those observed in routine clinical care -where injection frequency is generally lower- suggests that extended treatment intervals may be artificially prolonged, potentially compromising visual outcomes [7, 24–29]. Accordingly, careful evaluation of visual acuity outcomes is essential when interpreting treatment interval efficacy in real world settings. Importantly, our analysis demonstrated comparable VA improvements at both 12 and 24 months across eyes treated at extended intervals (Q12W-<Q16W and ≥Q16W intervals) and those treated at shorter intervals (≤Q8W and >Q8W-<Q12W), indicating that treatment decisions were appropriately guided by lesion activity across all treatment interval groups.

Our analysis revealed that approximately one-third of eyes (35.4%) failed to achieve treatment intervals of ≥8 weeks at 24 months, despite receiving intensive therapy. These findings are consistent with recent evidence reporting that 25% of patients maintained injection intervals of <6 weeks at 24 months, a group categorised as “high treatment burden” eyes [29]. In the same study, an additional 45% of eyes exhibited treatment intervals >6 and <12 weeks at 2 years, resulting in a combined 70% of patients (25 + 45 = 70%), comparable to the 53% observed with <12 week intervals in our cohort. Minor discrepancies between the two studies may be attributed to differences in cohort composition and methodology, including the broader international sample in the referenced study and our inclusion of multiple interval metrics (i.e. most frequent, maximum and final intervals) across a nationally representative dataset.

New generation anti-VEGF therapies are anticipated to prolong injection intervals while maintaining efficacy, thereby reducing treatment burden in nAMD patients. This benefit has already been demonstrated by faricimab [19]. In our series, ranibizumab was the most frequently administered agent, followed by aflibercept 2 mg, both of which have demonstrated favourable visual outcomes at 12 months in previous studies [12, 30–33]. Randomised clinical trials have shown that newer agents such as brolucizumab [34], faricimab [15], or aflibercept 8 mg [16, 35] achieve non-inferior visual acuity gains compared to aflibercept 2 mg every 8 weeks (Q8W), with extended dosing intervals up to every 16 weeks (Q16W). Furthermore, RWE suggests that intravitreal faricimab may reduce treatment burden in patients requiring frequent injections with conventional anti-VEGF therapies [19, 36]. To date, real-world evidence supports the clinical trial findings for faricimab in treatment-naïve patients with nAMD and DME, demonstrating both anatomical stability and extended durability [35]. Despite these advances, the implementation of newer agents faces several barriers, including the need for updated treatment protocols, clinical guideline revisions, resistance to change in clinical practice, and cost considerations. Therefore, evaluating outcomes with first-generation anti-VEGF therapies remains essential to quantify the current unmet needs and to better define their role within the evolving nAMD treatment landscape.

This study presents some limitations. The external validity of the results reported may be limited as participating centres represent a selection of tertiary referral academic centres and advanced private practices [13], which may not reflect the reality of nAMD management nationwide in Spain or other countries or regions. Differences in treatment practices, healthcare infrastructure, or populations should be considered when interpreting and comparing to other international cohorts. Additionally, other potential confounding variables difficult to address in real-world data may have influenced treatment intervals (i.e. the influence of treatment switch).

In conclusion, this study demonstrates that, despite injection frequency falling within the TAE framework, a substantial proportion of eyes fail to achieve extended treatment intervals at both 12 and 24 months. These findings provide precise estimates of the extent of the unmet therapeutic need in the management of nAMD with currently available first-generation anti-VEGF agents. This evidence is critical for informing clinical decision-making, increasing awareness among healthcare providers, and guiding the development and implementation of more effective strategies aimed at reducing treatment burden and preventing vision loss associated with nAMD.

## Summary

### What was known before

- Real-world anti-VEGF therapy outcomes in neovascular AMD are suboptimal compared to randomised clinical trials for a variety of reasons, that include the treatment of unselected study cohorts, the high treatment burden required and the limited duration of first-generation anti-VEGF drugs.
- The magnitude of the unmet need in terms of percentage of eyes not achieving extended treatment intervals with first generation anti-VEGF drugs is not well established in routine clinical care.
- It is critical to identify the size of this unmet need, as this information could help clinicians to direct treatment decisions and design management algorithms in the current therapeutic scenario, that encompasses biosimilars and new generation anti-VEGF drugs.

### What this study adds

- This real-world national database study reveals that a high percentage of eyes do not achieve extended treatment intervals (≥12 weeks) at 12 and 24 months, highlighting a significant unmet need in nAMD management with first-generation anti-VEGF drugs.
- These findings provide a strong rationale for implementing novel longer-lasting anti-VEGF therapies in clinical practice to reduce treatment burden while maintaining disease control in a significant percentage of eyes that fail to achieve extended treatment intervals nationwide.
- This data can inform discussions with stakeholders about potential benefits of integrating new treatments into clinical pathways, illustrating the limits of biosimilars in terms of treatment intervals and setting the scene to guide future research on strategies directed to improve nAMD management and patient outcomes.

## Supplementary information

Supplemental table 1

Supplemental table 2

Supplemental table 3

Supplemental table 4
