# Supplementary material for: Treatment intervals with first-generation anti-vascular endothelial growth factor drugs: evaluating the unmet need in a real-world neovascular age-related macular degeneration national database
Source: Eye (Lond). 2025 Oct 16;39(18):3306–13. doi: 10.1038/s41433-025-03996-8 (PMC12669756; doi:10.1038/s41433-025-03996-8)
Supplement: Supplementary file 1 — Supplemental table 1 [file 41433_2025_3996_MOESM1_ESM.pdf]

Supplementary Table 1: Demographic and clinical characteristics of the total number of eyes analyzed in the study.

Variable	nAMD
Eyes (n)	1950
Patients (n)	1629
Gender, % female patients	60
Age, mean (SD)	79.6 (7.8)
Smoking status, n (%)	
Active smoker	78 (4)
Non-smoker	444 (23)
Ex-smoker	136 (7)
Unknown	1292 (66)
Time from diagnosis (days), mean (SD)	0 (0.8)
Baseline VA, mean (SD)	56 (20.7)
≤35 letters, n (%)	365 (19)
≥70 letters, n (%)	632 (32)
Lesion type, n (%)	
Type 1	411 (21)
Type 2	290 (15)
Type 3	188 (10)
PCV	51 (3)
Mixed	17 (1)
Unknown	993 (51)
Initial injection, n (%)	
Aflibercept	808 (41)
Brolucizumab	0 (0)
Ranibizumab	1142 (59)

Abbreviations: SD = Standard deviation; VA = Visual acuity**.**
