# Supplementary material for: Treatment intervals with first-generation anti-vascular endothelial growth factor drugs: evaluating the unmet need in a real-world neovascular age-related macular degeneration national database
Source: Eye (Lond). 2025 Oct 16;39(18):3306–13. doi: 10.1038/s41433-025-03996-8 (PMC12669756; doi:10.1038/s41433-025-03996-8)
Supplement: Supplementary file 2 — Supplemental table 2 [file 41433_2025_3996_MOESM2_ESM.pdf]

Supplementary Table 2: Summary of treatment and disease burden at 12 and 24 months after anti-VEGF treatment initiation of the total number of eyes analyzed in the study.

Variable	12 Months	24 Months
Eyes (n)	1950	1161
Injections, mean (SD)	7 (2.1)	11.4 (3.8)
Injections, median (Q1, Q3)	7 (6, 8)	11 (9, 14)
Visits, mean (SD)	8.6 (2.2)	14.7 (4)
Visits, median (Q1, Q3)	8 (7, 10)	14 (12, 17)
Maximum treatment interval (days), median (Q1, Q3)	98 (77, 148)	139 (104, 233)
Most frequent treatment interval category, n (%)		
4 weeks	923 (47)	382 (33)
6 weeks	456 (23)	265 (23)
8 weeks	256 (13)	207 (18)
10 weeks	162 (8)	130 (11)
12 weeks	44 (2)	52 (4)
14 weeks	23 (1)	30 (3)
16 weeks	9 (0)	14 (1)
18+ weeks	77 (4)	81 (7)
Last treatment interval (days), median (Q1, Q3)	76 (56, 112)	85 (57, 140)
Last treatment interval category, n (%)		
4 weeks	121 (6)	73 (6)
6 weeks	220 (11)	115 (10)
8 weeks	305 (16)	139 (12)
10 weeks	340 (17)	151 (13)
12 weeks	273 (14)	135 (12)
14 weeks	165 (8)	97 (8)
16 weeks	67 (3)	89 (8)
18+ weeks	459 (24)	362 (31)
