# Supplementary material for: Treatment intervals with first-generation anti-vascular endothelial growth factor drugs: evaluating the unmet need in a real-world neovascular age-related macular degeneration national database
Source: Eye (Lond). 2025 Oct 16;39(18):3306–13. doi: 10.1038/s41433-025-03996-8 (PMC12669756; doi:10.1038/s41433-025-03996-8)
Supplement: Supplementary file 3 — Supplemental table 3 [file 41433_2025_3996_MOESM3_ESM.pdf]

Supplementary Table 3. Description of eyes achieving ≤Q8W, >Q8W-<Q12W, ≥Q12W-<Q16W, ≥Q16W intervals at 12 months.

	≤8 Weeks	>8 - <12 Weeks	≥12 - <16 Weeks	≥16 Weeks
Eyes (n)	502	288	372	116
Lesion type at baseline, n (%)				
Type 1	134 (27%)	72 (25%)	86 (23%)	24 (21%)
Type 2	75 (15%)	35 (12%)	70 (19%)	18 (16%)
Type 3	52 (10%)	28 (10%)	54 (15%)	16 (14%)
PCV	15 (3%)	12 (4%)	10 (3%)	2 (2%)
Mixed	5 (1%)	2 (1%)	3 (1%)	1 (1%)
Unknown	221 (44%)	139 (48%)	149 (40%)	55 (47%)
Baseline VA, mean (SD)	57 (19)	56.8 (18.7)	56.1 (20.3)	55.9 (21.3)
Final VA, mean (SD)	61.7 (18.7)	60.3 (19.1)	62.2 (20.3)	60.7 (19)
≤35 letters, % baseline / % final	16% / 12%	17% / 13%	19% / 13%	20% / 16%
≥70 letters, % baseline / % final	33% / 45%	28% / 44%	33% / 52%	34% / 44%
VA change, mean (95% CI)	4.7 (3.1, 6.2)	3.5 (1.6, 5.4)	6.1 (4.3, 7.9)	4.8 (1.8, 7.8)
Lesion activity, all visits				
Unknown	46%	48%	39%	41%
Inactive	19%	23%	37%	28%
Active with SRF only	13%	10%	7%	6%
Active (any combination of fluid excluding SRF only)	22%	20%	18%	24%
Lesion activity, at last visit				
Unknown	39%	35%	19%	14%
Inactive	30%	34%	54%	43%
Active with SRF only	14%	14%	10%	10%
Active (any combination of fluid excluding SRF only)	16%	17%	17%	33%
Injections, mean (SD)	8.9 (1.3)	7.9 (0.9)	7.3 (0.9)	6.8 (0.9)
Injections, median (Q1, Q3)	9 (8, 10)	8 (7, 8)	7 (7, 8)	7 (6, 7)
Visits, mean (SD)	10.1 (1.9)	9.1 (1.7)	8.4 (1.7)	8 (1.5)
Visits, median (Q1, Q3)	10 (9, 11)	9 (8, 10)	8 (7, 9)	8 (7, 9)
Maximum treatment interval, median (Q1, Q3)	70.5 (61, 91)	73 (70, 87)	91 (84, 98)	120 (112, 128)
Most frequent treatment interval category, n (%)				
4 weeks	251 (50%)	105 (36%)	177 (48%)	64 (55%)
6 weeks	160 (32%)	57 (20%)	92 (25%)	23 (20%)
8 weeks	84 (17%)	44 (15%)	43 (12%)	18 (16%)
10 weeks	7 (1%)	81 (28%)	23 (6%)	6 (5%)
12 weeks	0 (0%)	1 (0%)	25 (7%)	2 (2%)
14 weeks	0 (0%)	0 (0%)	12 (3%)	1 (1%)
16 weeks	0 (0%)	0 (0%)	0 (0%)	2 (2%)
18+ weeks	0 (0%)	0 (0%)	0 (0%)	0 (0%)
Last treatment interval, median (Q1, Q3)	47 (37, 56)	70 (63, 70)	85 (83, 92)	119.5 (112, 127.2)
Last treatment interval category, n (%)				
4 weeks	91 (18%)	0 (0%)	0 (0%)	0 (0%)
6 weeks	170 (34%)	0 (0%)	0 (0%)	0 (0%)

8 weeks	241 (48%)	0 (0%)	0 (0%)	0 (0%)
10 weeks	0 (0%)	288 (100%)	0 (0%)	0 (0%)
12 weeks	0 (0%)	0 (0%)	233 (63%)	0 (0%)
14 weeks	0 (0%)	0 (0%)	139 (37%)	0 (0%)
16 weeks	0 (0%)	0 (0%)	0 (0%)	49 (42%)
18+ weeks	0 (0%)	0 (0%)	0 (0%)	67 (58%)
Initial injection, n (%)				
Aflibercept	196 (39%)	135 (47%)	159 (43%)	50 (43%)
Brolucizumab	0 (0%)	0 (0%)	0 (0%)	0 (0%)
Ranibizumab	306 (61%)	153 (53%)	213 (57%)	66 (57%)
Most frequent injection, n (%)				
Aflibercept	229 (46%)	155 (54%)	161 (43%)	46 (40%)
Brolucizumab	0 (0%)	0 (0%)	0 (0%)	0 (0%)
Ranibizumab	273 (54%)	133 (46%)	211 (57%)	70 (60%)

Abbreviations: PCV = Polypoidal choroidal vasculopathy; SD = Standard deviation; SRF = Subretinal fluid; VA = Visual acuity**.**
