# Supplementary material for: Treatment intervals with first-generation anti-vascular endothelial growth factor drugs: evaluating the unmet need in a real-world neovascular age-related macular degeneration national database
Source: Eye (Lond). 2025 Oct 16;39(18):3306–13. doi: 10.1038/s41433-025-03996-8 (PMC12669756; doi:10.1038/s41433-025-03996-8)
Supplement: Supplementary file 4 — Supplemental table 4 [file 41433_2025_3996_MOESM4_ESM.pdf]

Supplementary Table 4. Description of eyes achieving ≤Q8W, >Q8W-<Q12W, ≥Q12W-<Q16W, ≥Q16W intervals at 24 months in the cohort of eyes that fulfilled the additional selection criteria.

	≤8 Weeks	>8 - <12 Weeks	≥12 - <16 Weeks	≥16 Weeks
Eyes (n)	197	98	158	104
Lesion type at baseline, n (%)				
Type 1	53 (27%)	38 (39%)	42 (27%)	18 (17%)
Type 2	31 (16%)	15 (15%)	24 (15%)	14 (13%)
Type 3	22 (11%)	8 (8%)	23 (15%)	18 (17%)
PCV	7 (4%)	3 (3%)	3 (2%)	7 (7%)
Mixed	1 (1%)	1 (1%)	0 (0%)	0 (0%)
Unknown	83 (42%)	33 (34%)	66 (42%)	47 (45%)
Baseline VA, mean (SD)	60.4 (17.1)	59 (17.4)	59.5 (16.4)	59.2 (18.6)
Final VA, mean (SD)	66.1 (15.2)	62.7 (18.6)	63.6 (19.8)	62.3 (19.1)
≤35 letters, % baseline / % final	9% / 7%	13% / 9%	12% / 14%	14% / 13%
≥70 letters, % baseline / % final	40% / 55%	34% / 51%	32% / 61%	38% / 53%
VA change, mean (95% CI)	5.8 (3.5, 8.1)	3.7 (-0.2, 7.6)	4.1 (1.2, 7)	3 (-0.3, 6.4)
Lesion activity, all visits				
Unknown	45%	37%	38%	38%
Inactive	18%	27%	35%	35%
Active with SRF only	17%	15%	9%	10%
Active (any combination of fluid excluding SRF only)	20%	20%	18%	18%
Lesion activity, at last visit				
Unknown	44%	23%	19%	15%
Inactive	25%	39%	50%	55%
Active with SRF only	16%	16%	13%	13%
Active (any combination of fluid excluding SRF only)	15%	21%	18%	16%
Injections, mean (SD)	15.7 (2.6)	13.8 (1.9)	12.6 (1.9)	11.8 (2)
Injections, median (Q1, Q3)	15 (14, 18)	14 (12, 15)	12 (11, 14)	11 (10.8, 13)
Visits, mean (SD)	17.9 (3.7)	16 (4)	14.7 (3.6)	14.1 (3.3)
Visits, median (Q1, Q3)	18 (15, 20)	15 (13, 17)	14 (12, 16)	14 (11, 16)
Maximum treatment interval, median (Q1, Q3)	93 (75, 114)	93 (84, 110.8)	99 (91, 117.5)	119 (112, 126)
Most frequent treatment interval category, n (%)				
4 weeks	67 (34%)	15 (15%)	37 (23%)	33 (32%)
6 weeks	69 (35%)	23 (23%)	28 (18%)	18 (17%)
8 weeks	49 (25%)	31 (32%)	28 (18%)	21 (20%)
10 weeks	9 (5%)	24 (24%)	32 (20%)	8 (8%)
12 weeks	2 (1%)	3 (3%)	20 (13%)	7 (7%)
14 weeks	1 (1%)	2 (2%)	13 (8%)	7 (7%)
16 weeks	0 (0%)	0 (0%)	0 (0%)	8 (8%)
18+ weeks	0 (0%)	0 (0%)	0 (0%)	2 (2%)
Last treatment interval, median (Q1, Q3)	45 (38, 56)	69 (64.2, 70)	90 (84, 96.5)	112 (112, 123.5)
Last treatment interval category, n (%)				
4 weeks	36 (18%)	0 (0%)	0 (0%)	0 (0%)

6 weeks	70 (36%)	0 (0%)	0 (0%)	0 (0%)
8 weeks	91 (46%)	0 (0%)	0 (0%)	0 (0%)
10 weeks	0 (0%)	98 (100%)	0 (0%)	0 (0%)
12 weeks	0 (0%)	0 (0%)	85 (54%)	0 (0%)
14 weeks	0 (0%)	0 (0%)	73 (46%)	0 (0%)
16 weeks	0 (0%)	0 (0%)	0 (0%)	64 (62%)
18+ weeks	0 (0%)	0 (0%)	0 (0%)	40 (38%)
Initial injection, n (%)				
Aflibercept	77 (39%)	41 (42%)	66 (42%)	47 (45%)
Brolucizumab	0 (0%)	0 (0%)	0 (0%)	0 (0%)
Ranibizumab	120 (61%)	57 (58%)	92 (58%)	57 (55%)
Most frequent injection, n (%)				
Aflibercept	117 (59%)	49 (50%)	78 (49%)	52 (50%)
Brolucizumab	0 (0%)	0 (0%)	0 (0%)	0 (0%)
Ranibizumab	80 (41%)	49 (50%)	80 (51%)	52 (50%)

Abbreviations: PCV = Polypoidal choroidal vasculopathy; SD = Standard deviation; SRF = Subretinal fluid; VA = Visual acuity.
